# Characterization of a Novel *Conus bandanus* Conopeptide Belonging to the M-Superfamily Containing Bromotryptophan

**DOI:** 10.3390/md12063449

**Published:** 2014-06-05

**Authors:** Bao Nguyen, Jean-Pierre Le Caer, Gilles Mourier, Robert Thai, Hung Lamthanh, Denis Servent, Evelyne Benoit, Jordi Molgó

**Affiliations:** 1 Neurobiology and Development Laboratory, Research Unit # 3294, Institute of Neurobiology Alfred Fessard # 2118, National Center for Scientific Research, Gif sur Yvette Cedex 91198, France; E-Mails: bao.nguyen@inaf.cnrs-gif.fr or nguyenbaocbp@yahoo.com (B.N.); lamthanh5hung@yahoo.fr (H.L.); Evelyne.Benoit@inaf.cnrs-gif.fr (E.B.); 2 Institute of Biotechnology and Environment, University of Nha Trang, Nha Trang, Khanh Hoa 57000, Vietnam; 3 Research Unit # 2301, Natural Product Chemistry Institute, National Center for Scientific Research, Gif sur Yvette Cedex 91198, France; E-Mail: jean-pierre.lecaer@icsn.cnrs-gif.fr; 4 Molecular Engineering of Proteins, Institute of Biology and Technology Saclay, Atomic Energy Commission, Gif sur Yvette Cedex 91191, France; E-Mails: gilles.mourier@cea.fr (G.M.); robert.thai@cea.fr (R.T.); denis.servent@cea.fr (D.S.)

**Keywords:** *Conus bandanus*, cone snail venom, mass spectrometry, BnIIID, bromotryptophan, γ-carboxy glutamate, post-translational modifications

## Abstract

A novel conotoxin (conopeptide) was biochemically characterized from the crude venom of the molluscivorous marine snail, *Conus bandanus* (Hwass in Bruguière, 1792), collected in the south-central coast of Vietnam. The peptide was identified by screening bromotryptophan from chromatographic fractions of the crude venom. Tandem mass spectrometry techniques were used to detect and localize different post-translational modifications (PTMs) present in the BnIIID conopeptide. The sequence was confirmed by Edman’s degradation and mass spectrometry revealing that the purified BnIIID conopeptide had 15 amino acid residues, with six cysteines at positions 1, 2, 7, 11, 13, and 14, and three PTMs: bromotryptophan, γ-carboxy glutamate, and amidated aspartic acid, at positions “4”, “5”, and “15”, respectively. The BnIIID peptide was synthesized for comparison with the native peptide. Homology comparison with conopeptides having the III-cysteine framework (–CCx_1_x_2_x_3_x_4_Cx_1_x_2_x_3_Cx_1_CC–) revealed that BnIIID belongs to the M-1 family of conotoxins. This is the first report of a member of the M-superfamily containing bromotryptophan as PTM.

## 1. Introduction

The peptides from the venom of cone snails (conotoxins or conopeptides) constitute a rich source of useful pharmacological tools and peptide probes for ion channels, transporters, and neurotransmitter receptors with a high degree of diversity, specificity, and potency [1,2,3]. Each *Conus* species is estimated to have a large number of species-specific conotoxins comprising cysteine poor-/rich-peptides [4,5,6]. Most mature peptides range between 8 and 40 amino acid residues with various cysteine-framework, and inter-cyteine variations in the number and kind of amino acids. Moreover, they have a high degree of post-translational modifications (PTMs) [7], whereby the modifications serve to create efficiently new conotoxin structures and pharmacological properties [4]. In addition to extensive disulphide linkages, which are a common feature of conotoxins, common PTMs include *C*-terminal amidation, proline hydroxylation, γ-carboxylation of glutamic acid, pyroglutamic acid, tyrosine sulfation, tryptophan bromination, and glycosylations [8,9,10,11]. Thus, there is an important role to entirely characterize the complement of mature peptides from cone snails.

In the search of new conopeptides from the venom of *Conus bandanus*, collected from the coast of Vietnam, we have found an unusual peptide containing bromine. We used tandem mass spectrometry to characterize the primary peptide sequence and localize the positions of tryptophan bromination, and γ-carboxylation of glutamic acid. The amidation of aspartic acid of the *C*-terminus was confirmed by the combination of theoretical mass calculation, Edman degradation, and homology comparison. We compared also the peptide sequence similarity of BnIIID to that of other conopeptides from *Conus marmoreus*, considered a close-relative interspecies with the molluscivorous *Conus bandanus* [12,13]. In addition, the BnIIID peptide was synthesized for comparison with the native peptide. This is the first report of an M-superfamily conopeptide containing a bromotryptophan.

## 2. Results and Discussion

### 2.1. Venom Fractionation and Purification

**Figure 1:**
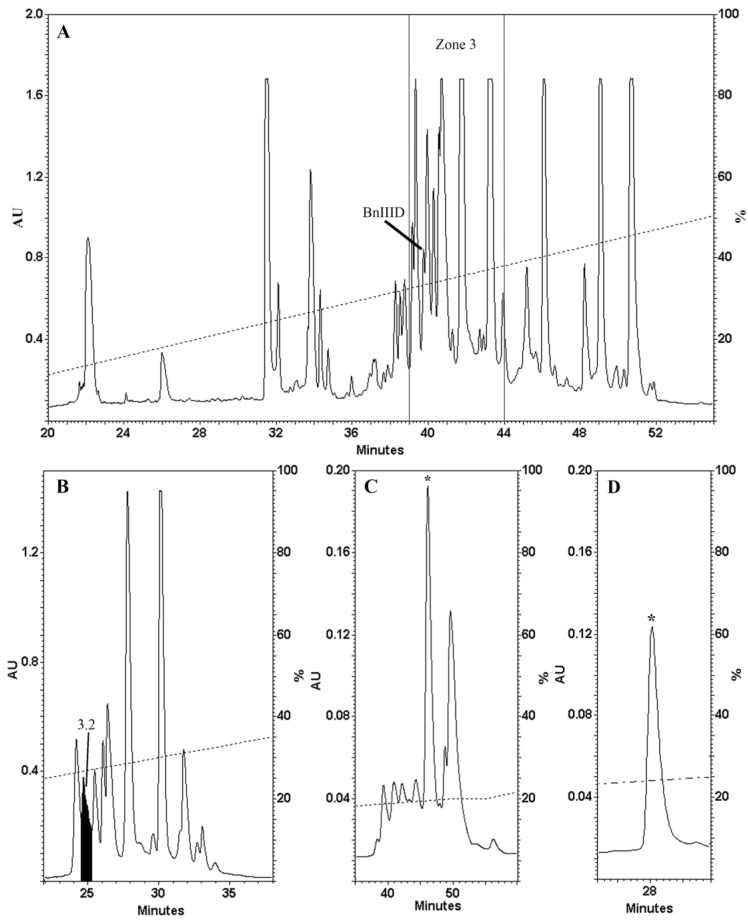
HPLC purification profile of dissected *C*. *bandanus* venom, extract, and BnIIID purification. (**A**) Fractionation was carried out with a Vydac semi-preparative C_18_ column (300 Å, 5 µm, 10 mm i.d. × 250 mm) and eluted at 3 mL min^−1^ with gradient 0%–56% of B buffer/50 min; (**B**) Fractionation from zone 3 with a Vydac analytical C_18_ column (300 Å, 5 µm, 4.6 mm i.d. × 250 mm) and eluted at 1 mL min^−1^ with gradient (0%–0.5% B/5 min, 0.5%–15% B/1 min and 15%–40% of B/40 min); (**C**) Further purification of fraction 3.2, from the previous step, was further purified with gradient (10%–15% B/7.5 min, 15%–20% B/42.5 min and 20%–35% B/45 min); (**D**) The asterisk indicates BnIIID. The purity of BnIIID peak was checked by MALDI-TOF-MS.

*C*. *bandanus* dissected crude venom was fractionated, sub-fractionated, and purified by reversed-phase chromatography on Vydac semi-preparative and analytical columns (Figure 1). Each HPLC fraction containing conopeptides (Figure 1A) was analyzed by Matrix-Assisted Laser Desorption/Ionization Time-Of-Flight (MALDI-TOF) mass spectrometry. The fraction 3.2 highlighted in black (Figure 1B) shows several different molecules with major intensity (Figure S1 in the Supplementary Information) containing an unusual monoisotopic ion. Figure 1C,D show further purifications from the previous fraction 3.2. An asterisk tags the subfraction that exhibited an unusual isotopic distribution for a peptide. This fraction was purified to near homogeneity on the same reversed-phase analytical column, by optimizing the elution gradient slope, hence, avoiding the use of a two dimensional chromatography (Figure 1D) and was named BnIIID.

### 2.2. Determination of the Number of Disulphide Bonds

**Figure 2:**
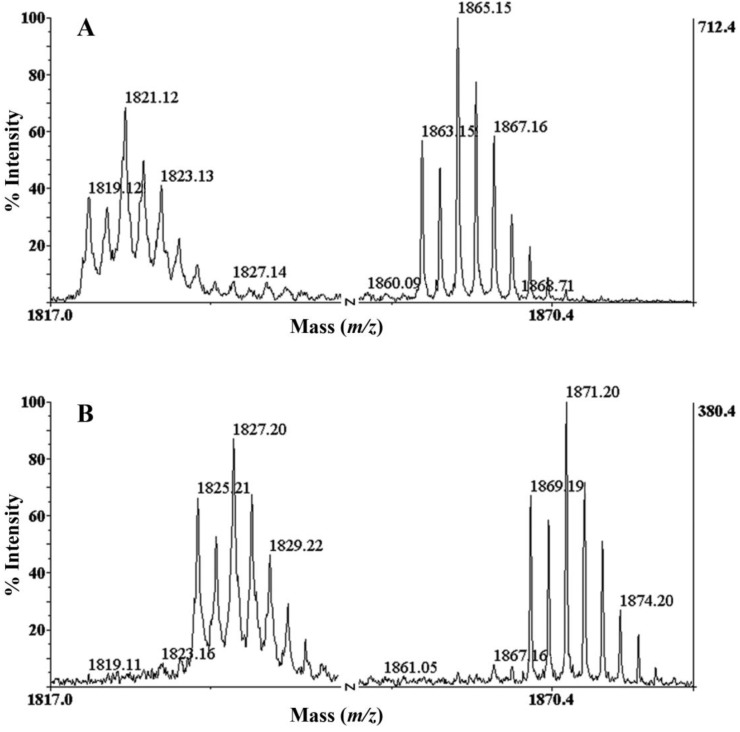
Identification of bromination using isotopic distributions of native BnIIID (**A**); and reduced BnIIID form (**B**); from off-line LC/MALDI-TOF MS. A shift of 6 u is observed characterizing the reduction of three S-S bonds.

Analysis of the native BnIIID by MALDI-TOF/TOF MS showed two single-charged ion distributions (Figure 2A), which yielded two monoisotopic ion signals at *m/z* 1819.129 and *m/z* 1863.159. The BnIIID isotopic distributions indicated the possible presence of one bromine atom [14,15]. The abundance of these peaks “doublet” at *m/z* (1819.129; 1821.21) and *m/z* (1863.159; 1865.159) is typical of a molecule containing the equally abundant bromine isotopes (50.69% ^79^Br and 49.31% ^81^Br, respectively). Following reduction with TCEP, BnIIID signals were shifted by 6 u indicating the presence of three disulfide bonds (Figure 2B).

### 2.3. Presence of Gamma-Carboxylate Glutamate Residue

**Figure 3:**
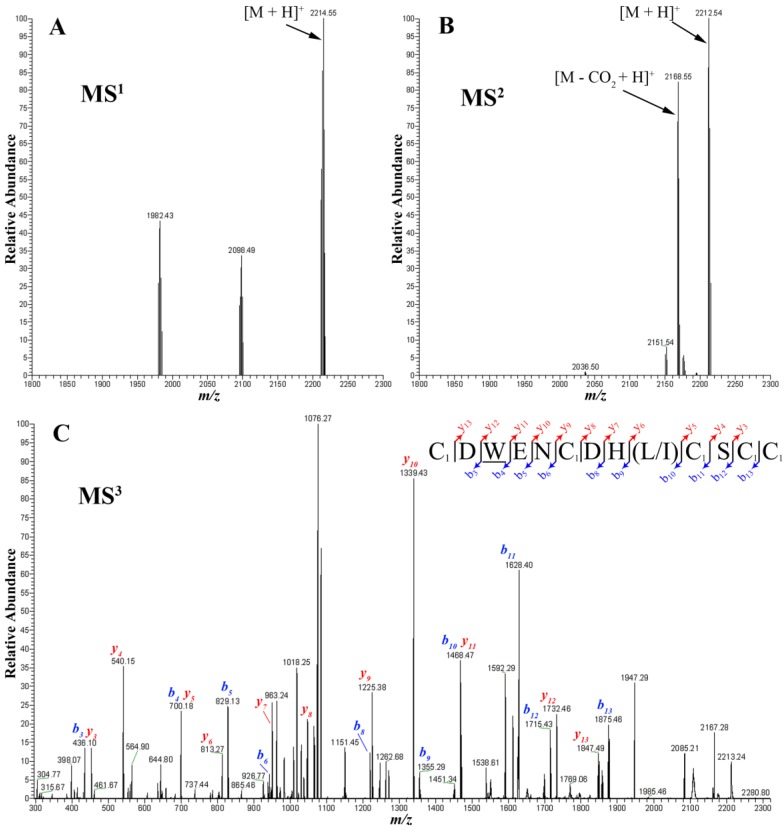
Analysis of reduced and alkylated BnIIID by high resolution MS and MSMS mass spectrometry: (**A**) Deconvoluted spectrum of the fraction shown in Figure 1D after reduction and alkylation with IAA; (**B**) Deconvoluted spectrum of the CID fragmentation of the 2214.55 Da ion; (**C**) MS^3^ product (2168.55 Da) corresponding to the decarboxylation of the precursor ion. Note: C_1_: carbamidomethyl-cysteine; alkylated cystein by IAA; W: bromotryptophan.

To elucidate the two different major masses of the pure fraction, we reduced and alkylated the cysteines to avoid disulfide reformation and to improve fragmentation yield during ESI-MSMS measurements (Figure 3). The CID fragmentation of the major compound in the fraction, 2214.55 Da, produce the decarboxylated fragment. In order to elucidate the sequence, the decarboxylated fragment was submitted to MS^3^ CID fragmentation. Figure 3C illustrate the *b* and *y* series obtained, confirming again the presence of a bromotryptophan. Thus, we could predict BnIIID having one γ-carboxylated glutamate residue and a bromotryptophan.

### 2.4. Peptide Sequencing

**Figure 4:**
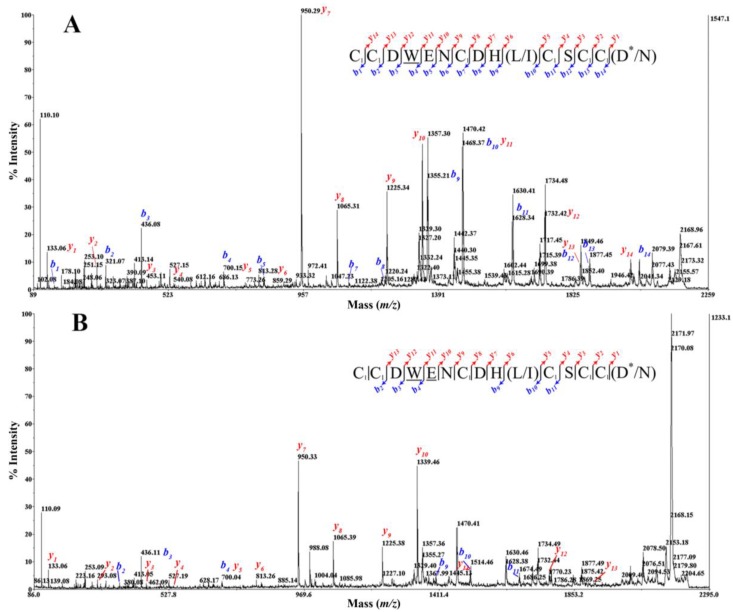
CID MS/MS spectrum of TCEP-reduced and IAA-labeled BnIIID, recorded with the MALDI-TOF/TOF 4800 mass spectrometer. MS/MS fragmentation of BnIIID without (**A**), and with carboxylation of E (**B**). Insets show the sequences derived from these MS/MS spectra. Note: C_1_: carbamidomethyl-cysteine; alkylated cystein by IAA; W: bromotryptophan; E: γ-carboxylic glutamic acid; *: *C*-terminal amidation.

To determine the amino acid sequence of BnIIID, we also employed the CID fragmentation technique using a MALDI TOF/TOF mass spectrometer that generated predominantly *b* and *y-*type product ions. Figure 4 shows two CID mass spectra of IAA-labeled BnIIID, with and without-carboxylation. Moreover, the product ions that contained a bromotryptophan residue showed unusual isotope patterns. The MS/MS spectrum of the parent ion at *m/z* 2167.245, without carboxylation (Figure 4A), showed complete series of *b* and *y-*type ions from position 1 to 15. We clearly observed a shift corresponding to the bromotryptophan residue (+260/262 u) between *b_3_* and *b_4_* ions or *y_11_* and *y_12_* ions. The glutamic acid residue is also identified after the bromotryptophan residue using the *b_4_/b_5_* and *y_10_/y_11_* ions. Furthermore, the *m/z* increment of 160 u between (*b_1_/b_2_*; *b_6_/b_7_*; *b_10_/b_11_*; *b_12_/b_13_*; *b_13_/b_14_*) ions confirmed the presence of an IAA-modified cysteine. Therefore, the mass of an IAA-modified cysteine at first position is inferred from *m/z* 161 *b_1_* ion and t corresponding *m/z* 2007 *y_14_* ion. Thus, BnIIID linear sequence exhibits a cysteine framework III, with the following pattern CC–C–C–CC. There are limits in distinguishing leucine/isoleucine residue (mass 113 Da) at position “10”, and amidated aspartic acid/asparagine residue (mass of 114 Da) at the *C*-terminus (*C*-terminal amidation is a common PTM due to 1 Da reduction in mass). Therefore, the initial sequence assignment of *m/z* 2167.245 parent ion was CCDWENCDH(L/I)CSCC(D*/N), as shown in Figure 4A.

**Figure 5:**
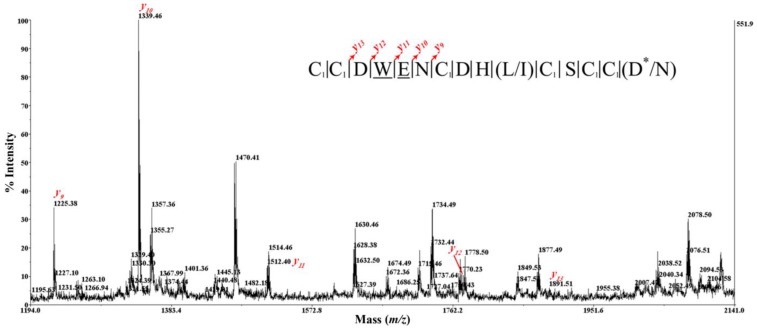
A zoom of CID MS/MS mass spectrum profile of IAA-labeled BnIIID with carboxylation of E (Figure 4B). Insets show the sequences derived from these MS/MS spectra. Note: C_1_: carbamidomethyl-cysteine; alkylated cystein by IAA; W: bromotryptophan; E: γ-carboxylic glutamic acid; *: *C*-terminal amidation.

Figure 4B shows the spectrum of the parent ion at *m/z* 2211.198 with predicted carboxylation. Here we focused on the identification of the γ-carboxylic glutamic acid residue. The *m/z* 2211.198- CID MS/MS spectrum is not so informative than the *m/z* 2167.245-spectrum due to the preferential loss of the carboxylic group of the γ-carboxylic glutamic acid residue during the fragmenting process. Though, we determined a γ-carboxy glutamate (E) according to the difference of 173 u at the same position “5”, *i*.*e*., between *y_10_* and *y_11_* ions (Figure 5). We also found a perfect agreement with ESI-MS/MS data using the series of *y-*type ions. Thus, the initial BnIIID sequence is CCDWENCDH(L/I)CSCC(D*/N) with two confirmed PTMs: bromotryptophan (W) and γ-carboxy glutamate (E). Nair *et al*. have used the Fourier transform ion cyclotron resonance MS/MS technique of electron capture dissociation, infrared multiphoton dissociation and CID to detect, and localize the bromotryptophan of a conopeptide, named Mo 1274, from *Conus monile* venom [16]. There was difficulty in the initial sequence assignment of CID mass spectrum of the Mo 1274 peptide. In our case, the MALDI-TOF CID MS/MS was used to characterize simply the initial sequence, and also positions of bromotryptophan and γ-carboxy glutamate.

**Figure 6:**
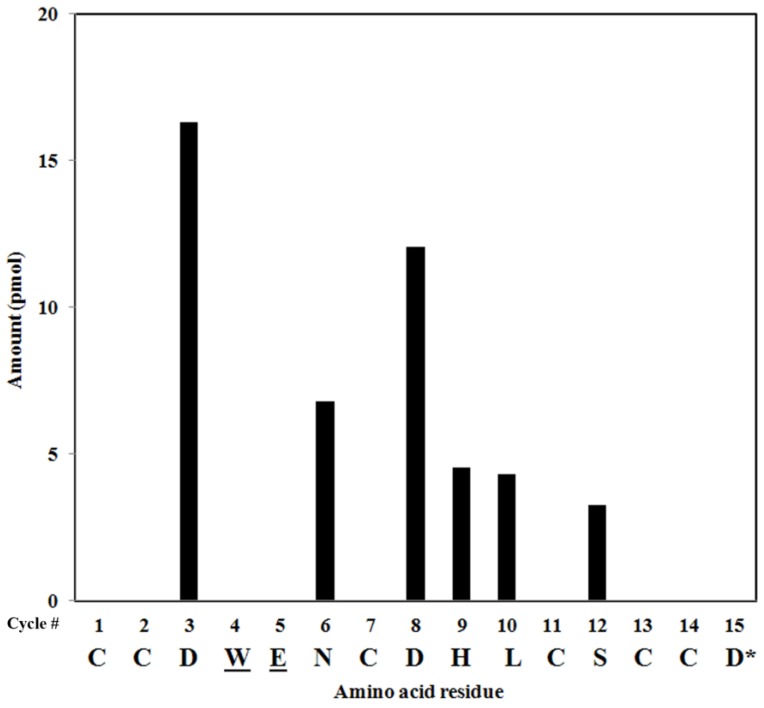
Solid-phase Edman degradation of native BnIIID. Note: W: bromotryptophan; E: γ-carboxylic glutamic acid; *: *C*-terminal amidation.

To confirm the complete amino acid sequence of BnIIID, this native conopeptide was submitted to Edman’s degradation. Figure 6 presents the release of BnIIID PTH-amino acid residues for each cycle. BnIIID sequence has totally 15 amino acid residues with six cysteines at positions “1; 2; 7; 11; 13; 14” which supports well the CID MS/MS obtained data. Additionally, there are no amounts of PTH-amino acid residues at cycle “4” and “5”, and it explains clearly that PTH-amino acid residue of bromotryptophan and γ-carboxy glutamate are not noted in Edman’s degradation profile of the standard program. At position “10”, we can confirm the leucine residue having an amount of 4.3 pmol in place of isoleucine. No data are observed at the last cycle, which may propose that there is a PTM in the amino acid residue. The MS/MS spectrum data and theoretical calculation offered previously two possibilities: an amidated aspartic acid (D*) or an asparagine (N) residue. Therefore, it may be inferred an aspartic acid amidation at the *C*-terminus. Thus, the complete linear BnIIID sequence is CCDWENCDHLCSCCD* with three PTMs: bromotryptophan, γ-carboxy glutamate, and amidated aspartic acid, at positions “4”, “5”, and “15”, respectively. This sequence is also in agreement with the experimental mass obtained in Figure 3B for BnIIID; theoretical mass of BnIIIID (C78 H111 N26 O33 S6 Br) = 2210.5309 Da, experimental mass: 2210.5293 Da (Δ = 1.6 ppm).

### 2.5. Peptide Synthesis

**Figure 7:**
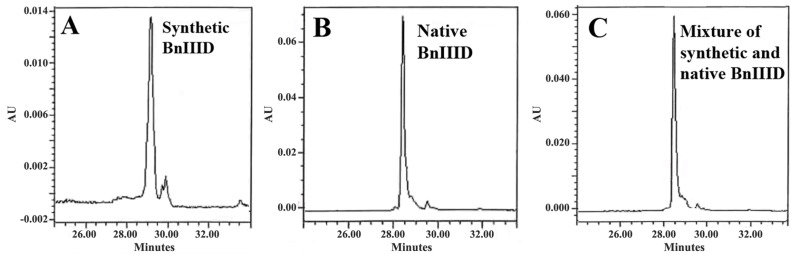
Verification of the synthetic peptide folding. The HPLC chromatograms of the synthetic BnIIID (**A**); the native peptide (**B**); and the co-elution of the mixture of synthetic and native BnIIID (**C**). The experiments were carried out with a X-Bridge analytical C_18_ column and eluted at 1 mL·min^−1^ with a gradient 0%–45% of acetonitrile in 40 min.

To confirm the refolding of the peptide like the native product, a synthetic BnIIID peptide was prepared (containing 6-d/l-6 bromotryptophan, γ-carboxy glutamate and amidated aspartic acid at positions “4”, “5”, and “15”, respectively). Figure 7 demonstrates the near identity of RP-HPLC chromatogram profiles from the same retention time, co-elution of refolded synthetic and native peptides. It is worth noting that the synthetic BnIIID was purified from the racemic mixture containing both d and l forms of 6-bromotryptophan. We deduced that the isomer position occurring in BnIIID is 6-bromotryptophan, following the earlier study by Craig *et al*. [14]. Remarkably, 6-bromotryptophan has also been found, mostly in other marine animals. The peptide component that co-eluted with the native peptide was less hydrophobic (data do not shown). This phenomenon is similar in the synthetic bromoheptapeptide purification of *Conus imperialis*, which demonstrated l-6-bromotryptophan from digestion with α-chymotrypsin [14]. Thus, it could be deduced that the native BnIIID contains l-6-bromotryptophan.

### 2.6. Sequence Similarity Analysis

As shown in Table 1, the BnIIID sequence was compared to other conopeptide sequences having the same “4/3/1”, III-cysteine framework (like –CCx_1_x_2_x_3_x_4_Cx_1_x_2_x_3_Cx_1_CC–) in three different *Conus* species (piscivorous, vermivorous, and molluscivorous) on the ConoServer [17]. The BnIIID peptide shares some homology with two conopeptides (S3-S01, S3-S02) of the piscivorous *Conus striatus*, and two other peptides (Ts3.6, LtIIID) of the vermivorous *Conus tessulatus* and *Conus litteratus*, respectively. Interestingly, BnIIID exhibits high homology with five *C*. *marmoreus* conopeptide sequences (e.g., Mr3.16, MrIIIE, MrIIIF, Mr3.18, and Mr3.8). The MrIIIE peptide of *C*. *marmoreus* has been shown to have (C1–C5, C2–C4, C3–C6) disulfide linkage arrangement using Edman analysis of the partially reduced peptide [18]. We suggest that the BnIIID conopeptide could share the same disulfide connectivity as the MrIIIE peptide. However, further work will be necessary to definitively attribute the disulfide linkage of BnIIID. All compared conopeptides presented in Table 1 belong to M-1 family of the M-superfamily. Thus, it can be inferred that BnIIID also belongs to the same family. Moreover, the bomotryptophan residue in BnIIID is found as a PTM for the first time, not only in a conopeptide having the cysteine framework III, but also in the M superfamily sequences.

Craig *et al*. (1997) [14] were the first to report the bromination of tryptophan residues in *Conus* venom components. The initial work was focused on two conopeptides: the bromosleeper conopeptide isolated from the piscivorous *Conus radiatus*, and a heptapeptide isolated from the vermivorous *Conus imperialis*. A mechanism for tryptophan bromination in cone snails has been proposed, such as the bromination of tryptophan to l-6-bromotryptophan by a bromo peroxidase [10,19,20]. The relative importance and the role of bromination in conopeptides are currently unknown.

**Table 1:** BnIIID sequence similarity with other conopeptides, from piscivorous (p), vermivorous (v), and molluscivorous (m) cone snails, belonging to the M superfamily. Note: W: bromotryptophan; E: γ-carboxylic glutamic acid; *: *C*-terminal amidation.

Name	Cone snail	Diet	Sequence																	Reference
S3-S01	*C*. *striatus*	p	-	C	C	P	K	E	W	C	N	R	D	C	S	C	C	T	-	[17,21]
S3-S02	*C*. *striatus*	p	-	C	C	P	A	R	M	C	M	A	A	C	S	C	C	D	-	[17,21]
Ts3.6	*C*. *tessulatus*	v	Q	C	C	D	W	Q	W	C	D	G	A	C	D	C	C	A	-	[22]
LtIIID	*C*. *litteratus*	v	-	C	C	D	W	E	W	C	D	E	L	C	S	C	C	W	-	[23]
Mr3.16	*C*. *marmoreus*	m	V	C	C	S	F	G	S	C	D	S	L	C	Q	C	C	D *	-	[5]
MrIIIE	*C*. *marmoreus*	m	V	C	C	P	F	G	G	C	H	E	L	C	Y	C	C	D *	-	[18]
MrIIIF	*C*. *marmoreus*	m	V	C	C	P	F	G	G	C	H	E	L	C	L	C	C	D *	-	[18]
Mr3.18	*C*. *marmoreus*	m	-	C	C	H	R	N	W	C	D	H	L	C	S	C	C	G	S	[5]
Mr3.8	*C*. *marmoreus*	m	-	C	C	H	W	N	W	C	D	H	L	C	S	C	C	G	S	[18]
BnIIID	*C*. *bandanus*	m	-	C	C	D	W	E	N	C	D	H	L	C	S	C	C	D *	-	This work

*Conus* are well-known for using many different strategies to capture their own preys through the use of a variety of conotoxins. The capacity to generate conopeptides, in which the number and nature of amino-acid residues can vary within putative inter-cysteines, is one of their most important and efficient strategies. To these characteristics it should be added that conopeptides may become still more diverse and complex considering the reported PTMs in *Conus* venoms [7,17]. Here, BnIIID was analyzed as belonging to the M-superfamily of conotoxins [18,21,24,25], which shares the same cysteine framework (–CC–C–C–CC–) pattern (Table 2). The conopeptide framework-III, according to available data, can be represented by µ-, ι-, ψ-, and κ-conotoxins, with pharmacological properties, such as blockage of voltage-gated sodium channels (µ-conotoxins), activation of voltage-gated sodium channels, without delaying channel inactivation (ι-conotoxins), modulation or blockage of nicotinic acetylcholine receptors (ψ-conotoxins), and blockage of voltage-gated potassium channels (κ-conotoxins). Studies are in progress to determine the molecular target(s) of BnIIID.

The –CCx_1_x_2_x_3_x_4_Cx_1_x_2_x_3_Cx_1_CC– conopeptides have been poorly explored for their specific pharmacological targets. The conopeptide LtIIIA from *C*. *litteratus*, among ~63 other peptides, was evaluated as belonging to the ι-conotoxin class [26]. Remarkably, this pattern exists mostly in mollusk- and worm-hunting cone snails (e.g., eight conopeptides in *C*. *marmoreus*). The synthesis of BnIIID conopeptide will enable us to investigate in detail its pharmacological properties, and, what is more important, the eventual role of the PTMs, since three other peptides (BnIIIA, BnIIIB, and BnIIIC) have been just isolated by us, and such peptides lack some of the PTMs (bromotrytophan and γ-carboxyglutamate) here reported.

**Table 2:** Inter-cysteine variation of “framework III” conopeptides *vs*. their pharmacological properties. Note: W: bromotryptophan; E: γ-carboxylic glutamic acid; *: *C*-terminal amidation; p: piscivorous; m: molluscivorous; v: vermivorous.

Name	Organism	Diet	Sequence																													Pharmacology	Reference
BuIIIA	*C*. *bullatus*	p	V	T	D	R	C	C	K	G	K	R	E	-	C	G	R	W	-	-	C	R	D	H	S	R	C	C *	-	-	-	µ-conotoxin	[27]
BuIIIB	*C*. *bullatus*	p	V	G	E	R	C	C	K	N	G	K	R	G	C	G	R	W	-	-	C	R	D	H	S	R	C	C *	-	-	-	µ-conotoxin	[27]
CIIIA	*C*. *catus*	p	-	-	G	R	C	C	E	G	P	N	G	-	C	S	S	R	W	-	C	K	D	H	A	R	C	C *	-	-	-	µ-conotoxin	[28]
CnIIIA	*C*. *consors*	p	-	-	G	R	C	C	D	V	P	N	A	-	C	S	G	R	W	-	C	R	D	H	A	Q	C	C *	-	-	-	µ-conotoxin	[28]
CnIIIB	*C*. *consors*	p	-	-	Z	G	C	C	G	E	P	N	L	-	C	F	T	R	W	-	C	R	N	N	A	R	C	C	R	Q	Q	µ-conotoxin	[28]
GIIIA	*C*. *geographus*	p	-	-	R	D	C	C	T	O	O	K	K	-	C	K	D	R	Q	-	C	K	O	Q	R	-	C	C	A*		-	µ-conotoxin	[29]
GIIIB	*C*. *geographus*	p	-	-	R	D	C	C	T	O	O	R	K	-	C	K	D	R	R	-	C	K	O	M	K	-	C	C	A*		-	µ-conotoxin	[30]
GIIIC	*C*. *geographus*	p	-	-	R	D	C	C	T	O	O	K	K	-	C	K	D	R	R	-	C	K	O	L	K	-	C	C	A*		-	µ-conotoxin	[31]
KIIIA	*C*. *kinoshitai*	p	-	-	-	-	C	C	N	-	-	-	-	-	C	S	S	K	W	-	C	R	D	H	S	R	C	C *	-	-	-	µ-conotoxin	[32]
LtIIIA	*C*. *litteratus*	v	-	-	D	E	C	C	E	O	Q	W	-	-	C	D	G	A	-	-	C	D	-	-	-	-	C	C	S	-	-	ι-conotoxin	[26]
MIIIA	*C*. *magus*	p	-	-	Z	G	C	C	N	V	P	N	G	-	C	S	G	R	W	-	C	R	D	H	A	Q	C	C *	-	-	-	µ-conotoxin	[33]
PIIIA	*C*. *purpurascens*	p	-	Z	R	L	C	C	G	F	O	K	S	-	C	R	S	R	Q	-	C	K	O	H	R	-	C	C *	-	-	-	µ-conotoxin	[34]
PIIIE	*C*. *purpurascens*	p	-	H	O	O	C	C	L	Y	G	K	-	-	C	R	R	Y	O	G	C	S	S	A	S	-	C	C	Q	R *		ψ-conotoxin	[35]
PIIIF	*C*. *purpurascens*	p	-	G	O	O	C	C	L	Y	G	S	-	-	C	R	O	F	O	G	C	Y	N	A	L	-	C	C	R	K *		ψ-conotoxin	[36]
PrIIIE	*C*. *parius*	p	-	A	A	R	C	C	T	Y	H	G	S	-	C	L	K	E	K	-	C	R	R	K	Y	-	C	C *	-	-	-	ψ-conotoxin	[37]
RIIIJ	*C*. *radiatus*	p	-	L	O	O	C	C	T	O	O	K	K	H	C	O	A	O	A	-	C	K	Y	K	O	-	C	C	K	S	-	κ-conotoxin	[38]
RIIIK	*C*. *radiatus*	p	-	L	O	S	C	C	S	L	N	L	R	L	C	O	V	O	A	-	C	K	R	N	O	-	C	C	T *		-	κ-conotoxin	[39]
SIIIA	*C*. *striatus*	p	-	-	Z	N	C	C	N	G	G	-	-	-	C	S	S	K	W	-	C	R	D	H	A	R	C	C *	-	-	-	µ-conotoxin	[32]
SIIIB	*C*. *striatus*	p	-	-	Z	N	C	C	N	G	G	-	-	-	C	S	S	K	W	-	C	K	G	H	A	R	C	C *	-	-	-	µ-conotoxin	[40]
SmIIIA	*C*. *stercusmuscarum*	p	-	-	Z	R	C	C	N	G	R	R	G	-	C	S	S	R	W	-	C	R	D	H	S	R	C	C	-	-	-	µ-conotoxin	[41]
SxIIIA	*C*. *striolatus*	p	-	-	-	R	C	C	T	G	K	K	G	S	C	S	G	R	A	-	C	K	N	L	K	-	C	C	A *		-	µ-conotoxin	[42]
SxIIIB	*C*. *striolatus*	p	-	-	Z	K	C	C	T	G	K	K	G	S	C	S	G	R	A	-	C	K	N	L	R	-	C	C	A *		-	µ-conotoxin	[42]
TIIIA	*C*. *tulipa*	p	-	R	H	G	C	C	K	G	O	K	G	-	C	S	S	R	E	-	C	R	O	Q	H	-	C	C *	-	-	-	µ-conotoxin	[43]
BnIIID	*C*. *bandanus*	m	-	-	-	-	C	C	D	W	E	N	-	-	C	D	H	L	-	-	C	S	-	-	-	-	C	C	D *		-	unknown	This work

## 3. Experimental Section

### 3.1. Isolation and Purification of Native Conopeptides

Twelve adult specimens (≥72 mm shell length) of *C*. *bandanus* were collected in the Nha Trang bay in the South central coast of Vietnam. We have used the same methods for venom extraction as previously described [13]. The extract was lyophilized and stored at −80 °C for later fractionation. All subfractions, purifications, and analyses were performed on a HPLC (System Gold with programmable solvent module 126 pumps using 32 Karat software; Beckman Coulter, Fullerton, CA, USA) at room temperature. Fractions were collected based on absorbance at 220 nm (System Gold 166 detector; Beckman Coulter, Fullerton, CA, USA). The elution buffers used for all HPLC were the following: A buffer (1000 mL H_2_O/1 mL trifluoroacetic acid (TFA; Merck, Darmstadt, Germany); B buffer (900 mL CH_3_CN/100 mL H_2_O/1 mL TFA). The lyophilized *C*. *bandanus* venom (~300 mg) was dissolved in A buffer, and the extract was loaded in batches of ~10 mg on a Vydac semi-preparative C_18_ column (300 Å, 5 µm, 10 mm i.d. × 250 mm). The gradient program for the semi-preparative column was 0% of B buffer/10 min, then 0%–56% of B buffer/50 min with the flow rate 3 mL/min. Further purification steps were carried out using a Vydac analytical C_18_ column (300 Å, 5 µm, 4.6 mm i.d. × 250 mm) at flow rate 1 mL/min.

### 3.2. Reduction-Alkylation Procedures

The purified fraction was reduced by incubation for 10 min at 65 °C in a solution of 20 mM tris-2-carboxyethyl-phosphine (TCEP) in 0.5 M HEPES. Alkylation was then performed by addition of iodoacetamide (IAA) 0.05 M and incubation for 20 min at room temperature in darkness. The mixture was finally desalted by solid phase extraction on a Zip Tip C18 column (Millipore, Billerica, MA, USA).

### 3.3. Mass Spectrometry Analysis

MALDI analysis—all bioactive fractions were analyzed with a 5800 MALDI-TOF/TOF mass spectrometer (AB Sciex, Les Ulis, France). The instrument was equipped with an Nd:YAG laser operating at 355 nm wavelength. Aliquots of 0.5 µL of a purified fraction were mixed with 0.5 µL of a solution of 4 mg/mL of cyano-4-hydroxycinnamic acid. Acquisitions were performed on positive reflection mode. Instrument calibration was done using a peptide mixt (peptide calibration 1 and 2 from ABSciex between 700 and 3700 Da). For MS/MS experiments, precursor ions were accelerated at 8 keV and the MS/MS spectra were acquired using 2 keV collision energy, with CID gas (air) at a pressure of 3.5 × 10^−6^ Torr. MS and MS/MS data were processed using DataExplorer 4.9 (AB Sciex).

Nano ESI orbitrap analysis—some important fractions were verified on their accurate mass on a LTQ Orbitrap mass spectrometer equipped with a nano-electrospray source (Thermo Scientific, Bremen, Germany). Few microliters of chromatographic fraction were loaded onto metal-coated borosilicate emitters (Thermo Scientific). The 1.2 KV were applied to the emitter and the acquisition was monitored on the orbitrap set with a theoretical resolution of 30,000 at *m*/*z* 400. For MS2 and MS3, normalized collision energy of 36 eV was applied. Spectra were treated with Xcalibur 2.1, the multiplied-charged species were recalculated into its singly-charged form using the Xtract software (Thermo Scientific, San Jose, CA, USA).

### 3.4. Automatic Amino Acid Sequencing

Pure peptide fractions were dissolved in buffer A and their concentration were measured by scan mode “Absorbance” (range 240–350 nm wavelength) in a DU 800 Spectrophotometer (Beckman-Coulter, Brea, CA, USA). The 2 µL native peptide (~190 µM) was sequenced using an automated Edman degradation using a Procise protein sequencer (Applied Biosystem model 492, Applied Biosystem, Foster City, CA, USA).

### 3.5. Chemical Synthesis

Materials—Fmoc-amino acids and 2-(6-Chloro-1-H-benzotriazole-1-yl)-1,1,3,3,-tetramethylaminium hexafluorophosphate (HCTU) were obtained from Activotec (Cambridge, UK), Fmoc-Gla(OtBu)2-OH from Iris Biotech (Marktredwitz, Germany) and Fmoc-dl-6-Bromotryptophan from AnaSpec (Fremont, CA, USA). The Chemmatrix resin and all the peptide synthesis grade reagents (*N*-methylpyrrolidone (NMP), *N*-methylmorpholine (NMM), piperidine, trifluoroacetic acid (TFA), anisole, thioanisole and triisopropylsilane (TPS) were purchased from Sigma Aldrich (Saint-Quentin Fallavier, France).

Synthesis—peptide synthesis was performed on a Protein Technologies, Inc Prelude synthesizer at a 50 µmole scale using fivefold excess of Fmoc-amino acid relative to the rink amide chemmatrix resin (0.4–0.6 mmol/g). All the amino acids were coupled twice 5 min using 1:1:2 amino acid/HCTU/NMM in *N*-methylpyrrolidone (NMP) with the exception of Fmoc-dl-6-bromotryptophane and Fmoc-Gla(OtBu)2-OH where a single 30 min coupling was used. Fmoc deprotection was performed using 20% piperidine in NMP, and NMP top washes were performed between deprotection and coupling steps. All cysteines were protected with S-trityl groups. Following chain assembly, the peptidyl-resin was treated with a mixture of TFA/thioanisole/anisole/TPS/water (82:5:5:2.5:5) for 2 h. The crude peptide was obtained after precipitation and washes in cold ethyl ether followed by dissolution in 10% acetic acid and lyophilisation.

Folding and characterization—refolding of the peptide was performed by stirring the crude reduced toxin in aqueous 0.1 M Tris buffer, pH 8.2 containing reduced and oxidized glutathione at 2 mM and 1 mM respectively for 24 and 3 h, respectively. The solution was then acidified using TFA 20% followed by purification by semi-preparative RP-HPLC using a X-Bridge (Waters, Milford, MA, USA) C_18_ column with a linear gradient from 0% to 60% buffer B in A at 4mL/min during 40 min, (buffer A, 0.1% TFA in water; Buffer B, acetonitrile, 0.1% TFA). The main fraction was collected and lyophilized. The homogeneity of the peptide was confirmed by MALDI-TOF mass spectrometry (AB SCIEX 4800, Les Ulis, France). The co-elution of the synthetic and native peptides was checked by analytical RP-HPLC.

## 4. Conclusions

In this work, we describe the characterization of a novel conopeptide named BnIIID, isolated from the venom of the molluscivorous snail species *C*. *bandanus* collected from the south central coast of Vietnam. The determination of the primary conopeptide structure on the basis of CID MS/MS mass-spectra analysis and Edman degradation revealed that BnIIID contained PTMs, such as bromotryptophan, γ-carboxy glutamate, and amidated aspartic acid at positions “4”, “5”, and “15”, respectively. The complete amino acid sequence of the conopeptide (CCDWENCDHLCSCCD*) and its III-cysteine framework allowed to categorize BnIIID in the M-1 family of conotoxins, belonging to the M-superfamily.

## Supplementary Files

Supplementary File 1 Supplementary Information (PDF, 187 KB)

## References

[1] Olivera B.M. (2006). *Conus* peptides: Biodiversity-based discovery and exogenomics. J. Biol. Chem..

[2] Lewis R.J., Dutertre S., Vetter I., Christie M.J. (2012). *Conus* venom peptide pharmacology. Pharmacol. Rev..

[3] Favreau P., Stocklin R. (2009). Marine snail venoms: Use and trends in receptor and channel neuropharmacology. Curr. Opin. Pharmacol..

[4] Olivera B.M.E.E. (1997). Just Lecture, 1996. *Conus* venom peptides, receptor and ion channel targets, and drug design: 50 million years of neuropharmacology. Mol. Biol. Cell.

[5] Dutertre S., Jin A.H., Kaas Q., Jones A., Alewood P.F., Lewis R.J. (2013). Deep venomics reveals the mechanism for expanded peptide diversity in cone snail venom. Mol. Cell. Proteomics.

[6] Terlau H., Olivera B.M. (2004). Conus venoms: A rich source of novel ion channel-targeted peptides. Physiol. Rev..

[7] Craig A.G., Bandyopadhyay P., Olivera B.M. (1999). Post-translationally modified neuropeptides from *Conus* venoms. Eur. J. Biochem..

[8] Jakubowski J.A., Kelley W.P., Sweedler J.V. (2006). Screening for post-translational modifications in conotoxins using liquid chromatography/mass spectrometry: An important component of conotoxin discovery. Toxicon.

[9] Gerwig G.J., Hocking H.G., Stocklin R., Kamerling J.P., Boelens R. (2013). Glycosylation of conotoxins. Mar. Drugs.

[10] Buczek O., Bulaj G., Olivera B.M. (2005). Conotoxins and the posttranslational modification of secreted gene products. Cell. Mol. Life Sci..

[11] Yates J.R. (2000). Mass spectrometry. From genomics to proteomics. Trends Genet..

[12] Nam H.H., Corneli P.S., Watkins M., Olivera B., Bandyopadhyay P. (2009). Multiple genes elucidate the evolution of venomous snail-hunting *Conus* species. Mol. Phylogenet. Evol..

[13] Nguyen B., Molgó J., Lamthanh H., Benoit E., Khuc T.A., Ngo D.N., Nguyen N.T., Millares P., le Caer J.P. (2013). High accuracy mass spectrometry comparison of *Conus bandanus* and *Conus marmoreus* venoms from the South Central Coast of Vietnam. Toxicon.

[14] Craig A.G., Jimenez E.C., Dykert J., Nielsen D.B., Gulyas J., Abogadie F.C., Porter J., Rivier J.E., Cruz L.J., Olivera B.M. (1997). A novel post-translational modification involving bromination of tryptophan. Identification of the residue, l-6-bromotryptophan, in peptides from *Conus imperialis* and *Conus radiatus* venom. J. Biol. Chem..

[15] England L.J., Imperial J., Jacobsen R., Craig A.G., Gulyas J., Akhtar M., Rivier J., Julius D., Olivera B.M. (1998). Inactivation of a serotonin-gated ion channel by a polypeptide toxin from marine snails. Science.

[16] Nair S.S., Nilsson C.L., Emmett M.R., Schaub T.M., Gowd K.H., Thakur S.S., Krishnan K.S., Balaram P., Marshall A.G. (2006). *De novo* sequencing and disulfide mapping of a bromotryptophan-containing conotoxin by Fourier transform ion cyclotron resonance mass spectrometry. Anal. Chem..

[17] Kaas Q., Yu R., Jin A.H., Dutertre S., Craik D.J. (2012). ConoServer: updated content, knowledge, and discovery tools in the conopeptide database. Nucleic Acids Res..

[18] Han Y.H., Wang Q., Jiang H., Liu L., Xiao C., Yuan D.D., Shao X.X., Dai Q.Y., Cheng J.S., Chi C.W. (2006). Characterization of novel M-superfamily conotoxins with new disulfide linkage. FEBS J..

[19] Jimenez E.C., Craig A.G., Watkins M., Hillyard D.R., Gray W.R., Gulyas J., Rivier J.E., Cruz L.J., Olivera B.M. (1997). Bromocontryphan: Post-translational bromination of tryptophan. Biochemistry.

[20] Jimenez E.C., Watkins M., Olivera B.M. (2004). Multiple 6-bromotryptophan residues in a sleep-inducing peptide. Biochemistry.

[21] Zhou M., Wang L., Wu Y., Zhu X., Feng Y., Chen Z., Li Y., Sun D., Ren Z., Xu A. (2013). Characterizing the evolution and functions of the M-superfamily conotoxins. Toxicon.

[22] Conticello S.G., Gilad Y., Avidan N., Ben-Asher E., Levy Z., Fainzilber M. (2001). Mechanisms for evolving hypervariability: The case of conopeptides. Mol. Biol. Evol..

[23] Pi C., Liu J., Peng C., Liu Y., Jiang X., Zhao Y., Tang S., Wang L., Dong M., Chen S., Xu A. (2006). Diversity and evolution of conotoxins based on gene expression profiling of *Conus litteratus*. Genomics.

[24] Corpuz G.P., Jacobsen R.B., Jimenez E.C., Watkins M., Walker C., Colledge C., Garrett J.E., McDougal O., Li W., Gray W.R. (2005). Definition of the M-conotoxin superfamily: Characterization of novel peptides from molluscivorous *Conus* venoms. Biochemistry.

[25] Jacob R.B., McDougal O.M. (2010). The M-superfamily of conotoxins: A review. Cell. Mol. Life Sci..

[26] Wang L., Liu J., Pi C., Zeng X., Zhou M., Jiang X., Chen S., Ren Z., Xu A. (2009). Identification of a novel M-superfamily conotoxin with the ability to enhance tetrodotoxin sensitive sodium currents. Arch. Toxicol..

[27] Holford M., Zhang M.M., Gowd K.H., Azam L., Green B.R., Watkins M., Ownby J.P., Yoshikami D., Bulaj G., Olivera B.M. (2009). Pruning nature: Biodiversity-derived discovery of novel sodium channel blocking conotoxins from *Conus bullatus*. Toxicon.

[28] Zhang M.M., Fiedler B., Green B.R., Catlin P., Watkins M., Garrett J.E., Smith B.J., Yoshikami D., Olivera B.M., Bulaj G. (2006). Structural and functional diversities among mu-conotoxins targeting TTX-resistant sodium channels. Biochemistry.

[29] Wakamatsu K., Kohda D., Hatanaka H., Lancelin J.M., Ishida Y., Oya M., Nakamura H., Inagaki F., Sato K. (1992). Structure-activity relationships of mu-conotoxin GIIIA: Structure determination of active and inactive sodium channel blocker peptides by NMR and simulated annealing calculations. Biochemistry.

[30] Hill J.M., Alewood P.F., Craik D.J. (1996). Three-dimensional solution structure of mu-conotoxin GIIIB, a specific blocker of skeletal muscle sodium channels. Biochemistry.

[31] Cruz L.J., Gray W.R., Olivera B.M., Zeikus R.D., Kerr L., Yoshikami D., Moczydlowski E. (1985). *Conus geographus* toxins that discriminate between neuronal and muscle sodium channels. J. Biol. Chem..

[32] Bulaj G., West P.J., Garrett J.E., Watkins M., Zhang M.M., Norton R.S., Smith B.J., Yoshikami D., Olivera B.M. (2005). Novel conotoxins from *Conus striatus* and *Conus kinoshitai* selectively block TTX-resistant sodium channels. Biochemistry.

[33] Wilson M.J., Yoshikami D., Azam L., Gajewiak J., Olivera B.M., Bulaj G., Zhang M.M. (2011). mu-Conotoxins that differentially block sodium channels NaV1.1 through 1.8 identify those responsible for action potentials in sciatic nerve. Proc. Natl. Acad. Sci. USA.

[34] Shon K.J., Olivera B.M., Watkins M., Jacobsen R.B., Gray W.R., Floresca C.Z., Cruz L.J., Hillyard D.R., Brink A., Terlau H. (1998). mu-Conotoxin PIIIA, a new peptide for discriminating among tetrodotoxin-sensitive Na channel subtypes. J. Neurosci..

[35] Shon K.J., Grilley M., Jacobsen R., Cartier G.E., Hopkins C., Gray W.R., Watkins M., Hillyard D.R., Rivier J., Torres J. (1997). A noncompetitive peptide inhibitor of the nicotinic acetylcholine receptor from *Conus purpurascens* venom. Biochemistry.

[36] Van Wagoner R.M., Jacobsen R.B., Olivera B.M., Ireland C.M. (2003). Characterization and three-dimensional structure determination of psi-conotoxin Piiif, a novel noncompetitive antagonist of nicotinic acetylcholine receptors. Biochemistry.

[37] Lluisma A.O., Lopez-Vera E., Bulaj G., Watkins M., Olivera B.M. (2008). Characterization of a novel psi-conotoxin from *Conus parius* Reeve. Toxicon.

[38] Chen P., Dendorfer A., Finol-Urdaneta R.K., Terlau H., Olivera B.M. (2010). Biochemical characterization of kappaM-RIIIJ, a Kv1.2 channel blocker: evaluation of cardioprotective effects of kappaM-conotoxins. J. Biol. Chem..

[39] Ferber M., Sporning A., Jeserich G., DeLaCruz R., Watkins M., Olivera B.M., Terlau H. (2003). A novel *Conus* peptide ligand for K^+^ channels. J. Biol. Chem..

[40] Schroeder C.I., Ekberg J., Nielsen K.J., Adams D., Loughnan M.L., Thomas L., Adams D.J., Alewood P.F., Lewis R.J. (2008). Neuronally micro-conotoxins from *Conus striatus* utilize an alpha-helical motif to target mammalian sodium channels. J. Biol. Chem..

[41] West P.J., Bulaj G., Garrett J.E., Olivera B.M., Yoshikami D. (2002). Mu-conotoxin SmIIIA, a potent inhibitor of tetrodotoxin-resistant sodium channels in amphibian sympathetic and sensory neurons. Biochemistry.

[42] Walewska A., Skalicky J.J., Davis D.R., Zhang M.M., Lopez-Vera E., Watkins M., Han T.S., Yoshikami D., Olivera B.M., Bulaj G. (2008). NMR-based mapping of disulfide bridges in cysteine-rich peptides: Application to the mu-conotoxin SxIIIA. J. Am. Chem. Soc..

[43] Lewis R.J., Schroeder C.I., Ekberg J., Nielsen K.J., Loughnan M., Thomas L., Adams D.A., Drinkwater R., Adams D.J., Alewood P.F. (2007). Isolation and structure-activity of mu-conotoxin TIIIA, a potent inhibitor of tetrodotoxin-sensitive voltage-gated sodium channels. Mol. Pharmacol..

