# Supplementary material for: Characterization of a Novel Conus bandanus Conopeptide Belonging to the M-Superfamily Containing Bromotryptophan
Source: Mar Drugs. 2014 Jun 5;12(6):3449–65. doi: 10.3390/md12063449 (PMC4071585; doi:10.3390/md12063449)
Supplement: Supplementary File 1 — Supplementary Information (PDF, 187 KB) [file marinedrugs-12-03449-s001.pdf]

Supplementary Information

Figure S1. The MALDI-TOF spectra of the fraction “3.2” highlighted in black in Figure 1B.

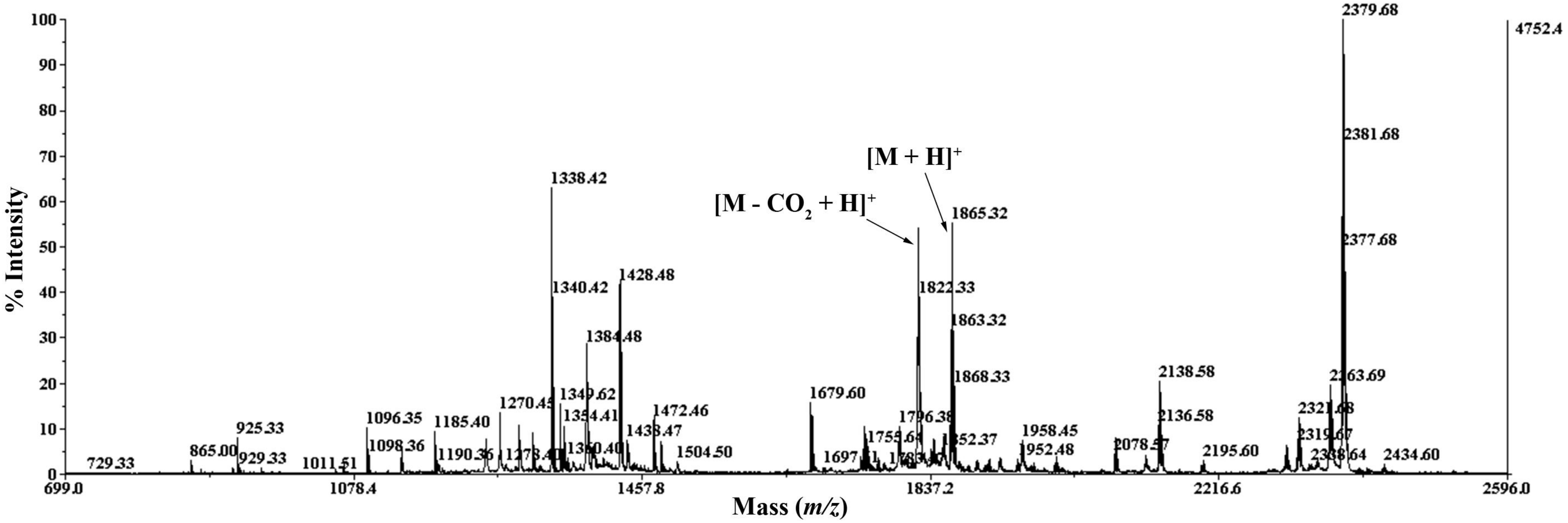

© 2014 by the authors; licensee MDPI, Basel, Switzerland. This article is an open access article distributed under the terms and conditions of the Creative Commons Attribution license (http://creativecommons.org/licenses/by/3.0/).
